# A Case of Neonatal Hypomelanosis of Ito With Early-Onset Refractory Seizures and Cortical Malformations

**DOI:** 10.7759/cureus.97298

**Published:** 2025-11-19

**Authors:** Yahya M Alassouli, Saja Tahir, Sukanya Vrushabhendra, Pawan Kashyape, Manigandan Thyagarajan, Mohamed O E Babiker

**Affiliations:** 1 Pediatric Neurology, Al Jalila Children's Speciality Hospital, Dubai, ARE; 2 Diagnostic Radiology, Al Jalila Children's Speciality Hospital, Dubai, ARE

**Keywords:** dermatology, early seizure, hypomelanosis, hypomelanosis of ito, neonate, neuro-cutaneous, neurology, neuro-radiology, pediatrics, refractory seizure

## Abstract

Hypomelanosis of Ito (HI) is a rare neurocutaneous syndrome characterized by unilateral or bilateral hypopigmented skin lesions arranged in linear, whorled, or patchy patterns, typically following the lines of Blaschko. The condition is frequently associated with multisystem involvement, including abnormalities of the central nervous system, musculoskeletal structures, ocular system, and dentition. We report the case of a seven-day-old female neonate who presented with left-sided hypopigmented skin lesions accompanied by ipsilateral hemihypertrophy and focal clonic seizures of the left upper and lower limbs. Physical examination revealed well-demarcated, linear, and whorled hypopigmented macules distributed along the lines of Blaschko, confined to the left side of the trunk and extremities while sparing the face. Dysmorphic features included macrocephaly, frontal bossing, deep-set eyes with epicanthic folds, low-set ears, and micrognathia. Neurological examination demonstrated generalized hypotonia with diminished neonatal reflexes. Although rare, HI is acknowledged as the third most prevalent neurocutaneous syndrome. We report one of the earliest documented neonatal presentations, manifesting with focal seizures within the first week of life. The case highlights the hallmark dermatological and neuroimaging features of HI and is distinguished by the rare coexistence of multiple radiological abnormalities. This study underscores the critical role of early neuroimaging in neonates presenting with cutaneous mosaicism and seizures, enabling timely recognition of underlying structural brain anomalies.

## Introduction

Hypomelanosis of Ito (HI) refers to a group of neuroectodermal disorders characterized by cutaneous features, including multiple hypopigmented streaks or whorls that follow the lines of Blaschko. It has been reported in approximately one in 3000 to one in 10,000 children [1]. Typically, the condition is accompanied by extracutaneous involvement, mainly affecting the neurological and musculoskeletal systems [1]. The exact cause remains unknown; however, many cases have been linked to genetic mosaicism and sporadic gene mutations. Recent studies have identified mosaic mechanistic target of rapamycin (mTOR) variants as a genetic basis for some cases of HI, linking the characteristic skin mosaicism with neurodevelopmental issues such as seizures and cortical malformations [2]. Diagnosis is mainly clinical, based on recognizing distinctive skin features, associated extracutaneous involvement, and supportive imaging findings [3]. Currently, there is no definitive cure for HI, and dermatologic treatment is usually not necessary. Nevertheless, cosmetic procedures may be recommended to address psychosocial concerns [4]. Management involves a multidisciplinary approach, emphasizing early detection and treatment of related extracutaneous symptoms [4]. This report highlights an early neonatal presentation of HI with focal refractory seizures and uncommon radiological features, underscoring the importance of early neuroimaging in evaluating complex neonatal seizures.

## Case presentation

A seven-day-old female neonate was referred for evaluation of recurrent focal seizures. The mother was a known case of type 2 diabetes mellitus. An antenatal ultrasound had demonstrated moderate to severe unilateral ventriculomegaly and dysgenesis of the corpus callosum. The infant was delivered via elective lower segment cesarean section (LSCS) at 35+5 weeks of gestation, with a good Apgar score. Birth weight was 3750 g, height 49 cm, and head circumference 36 cm. No antenatal steroids were given to the mother. 

The baby was admitted to the NICU for respiratory distress and an abnormal antenatal scan. She was comforted in an incubator and kept under cardio-respiratory monitoring. In view of macrocephaly and antenatal findings of ventriculomegaly, a brain ultrasound scan was done on the first day of life, which revealed midline brain abnormalities, asymmetry of the brain hemispheres, absent septum pellucidum, and agenesis of the corpus callosum.

The first neurological manifestation occurred on day seven of life, when the neonate exhibited focal clonic seizures involving the left upper and lower limbs. Feeding difficulties were also noted. She was initially treated with a loading dose of phenobarbitone (20 mg/kg), followed by maintenance therapy (4 mg/kg/day).

**Figure 1:**
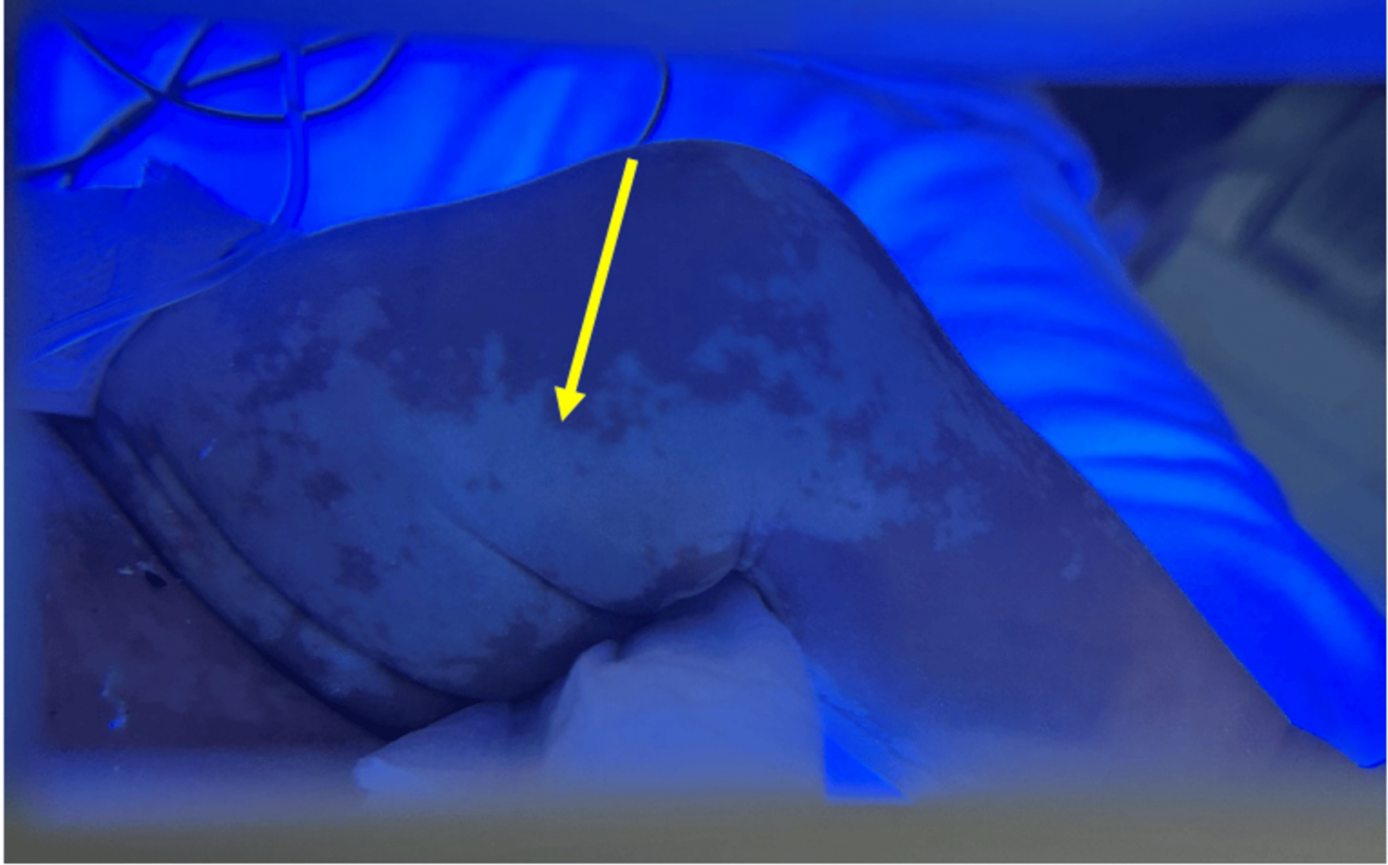
Wood's lamp showing enhancement of the hypo-pigmented lesions on the left lower limb.

On physical examination, the neonate had multiple hypopigmented macules distributed along the lines of Blaschko, predominantly on the left side of the body, involving the trunk and limbs, without crossing the midline. These lesions were more apparent under Wood's lamp illumination (Figure 1). 

Dysmorphic features included macrocephaly, frontal bossing, deep-set eyes with epicanthic folds, low-set ears, micrognathia, and left-sided hemihypertrophy. Ophthalmologic assessment revealed bilateral engorged retinal blood vessels with hypopigmented fundi. Neurological examination demonstrated generalized hypotonia and poorly elicitable neonatal reflexes.

**Figure 2:**
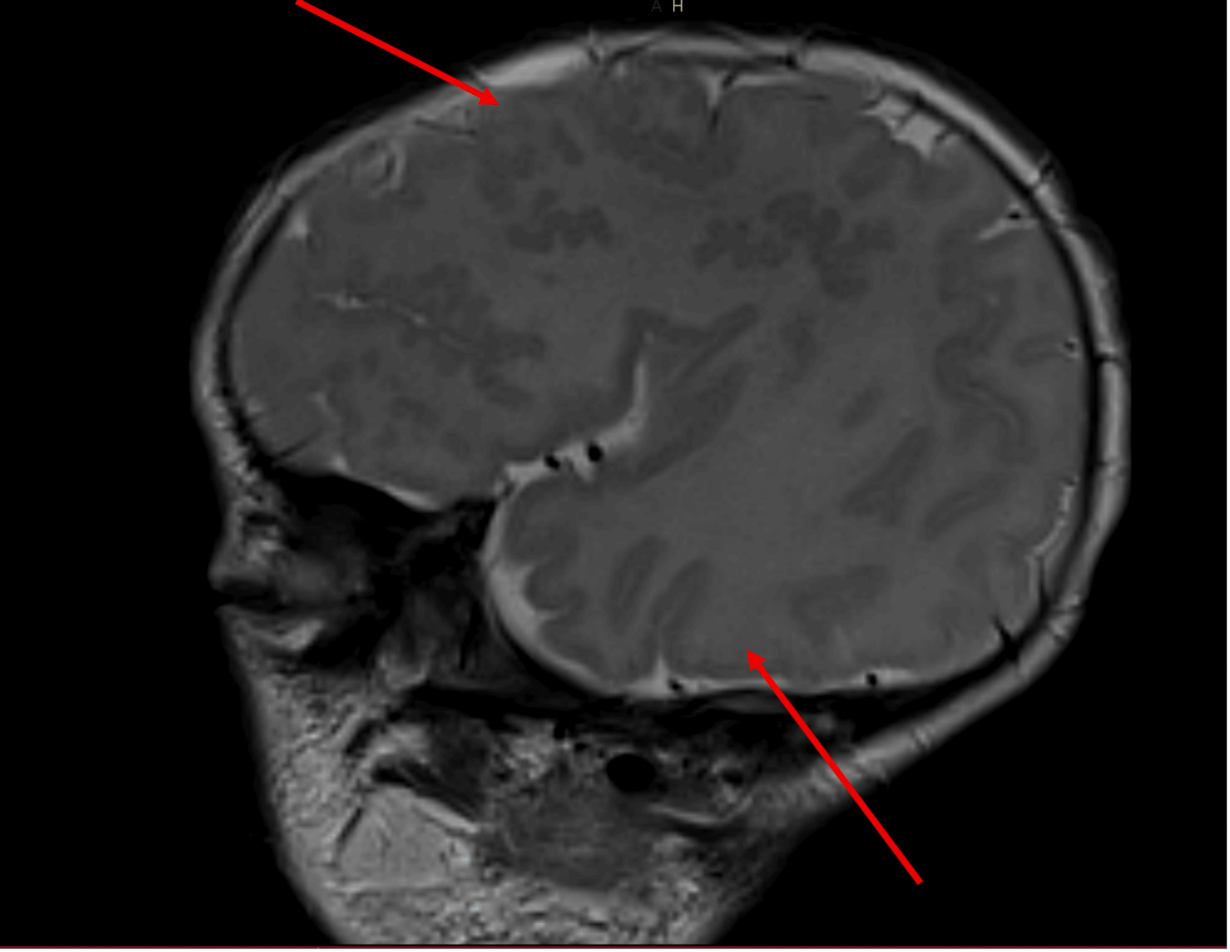
T2 sagittal sequence of the brain demonstrating lissencephaly and polymicrogyria.

**Figure 3:**
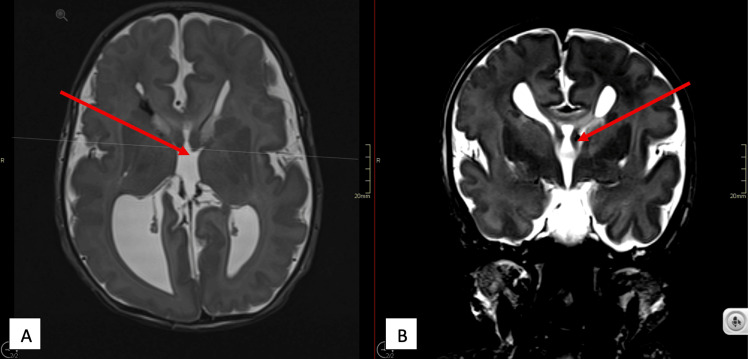
T2 axial (A) and T2 coronal (B) images of the brain demonstrating absent septum pellucidum.

**Figure 4:**
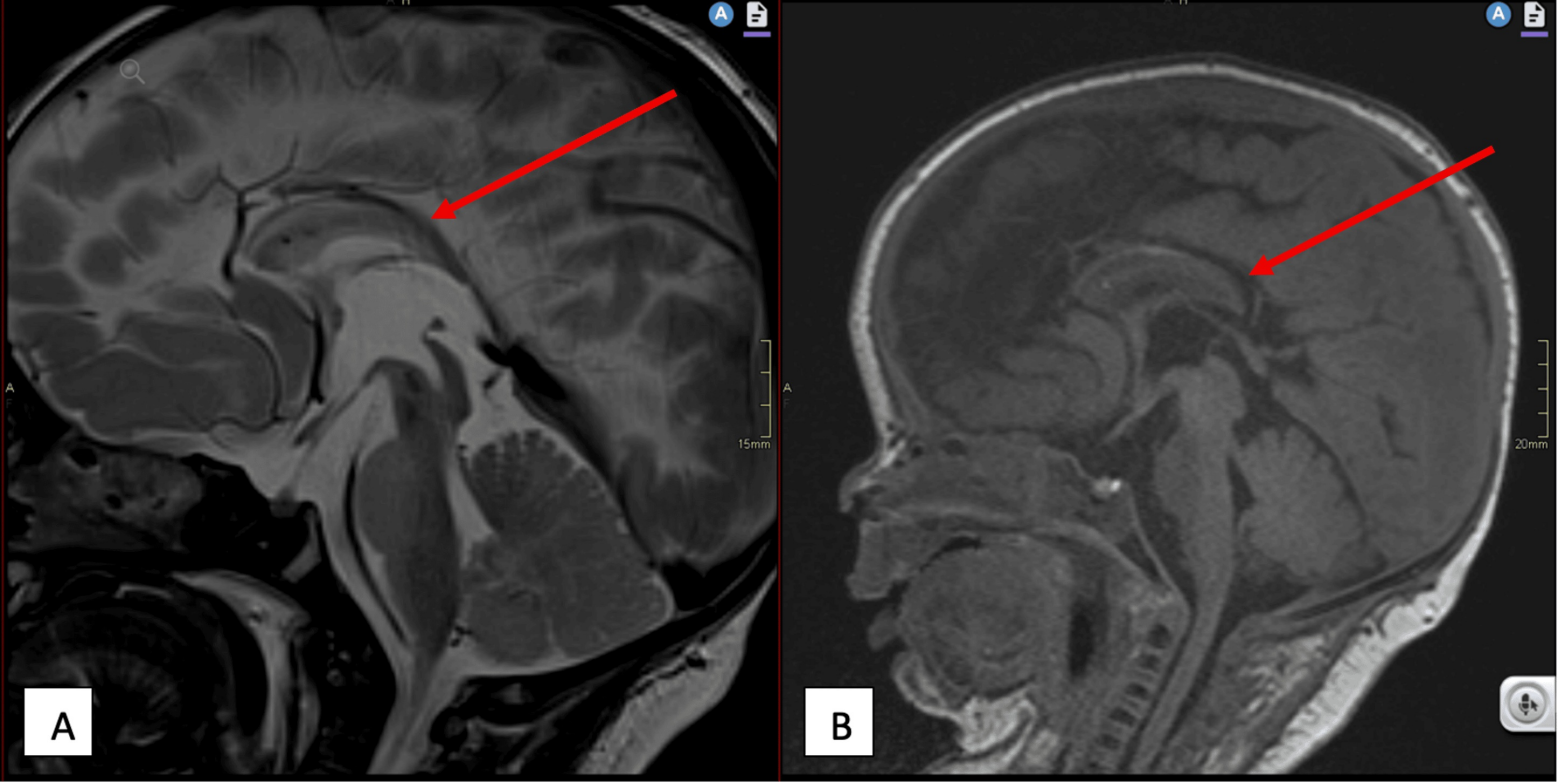
T2 (A) and T1 (B) sagittal sequences of the brain demonstrating a hypoplastic corpus callosum.

Sepsis screening yielded negative results. Brain MRI revealed bilateral sylvian polymicrogyria, gray matter heterotopia, lissencephaly, absence of the septum pellucidum, and a hypoplastic corpus callosum (Figures 2-4).

**Figure 5:**
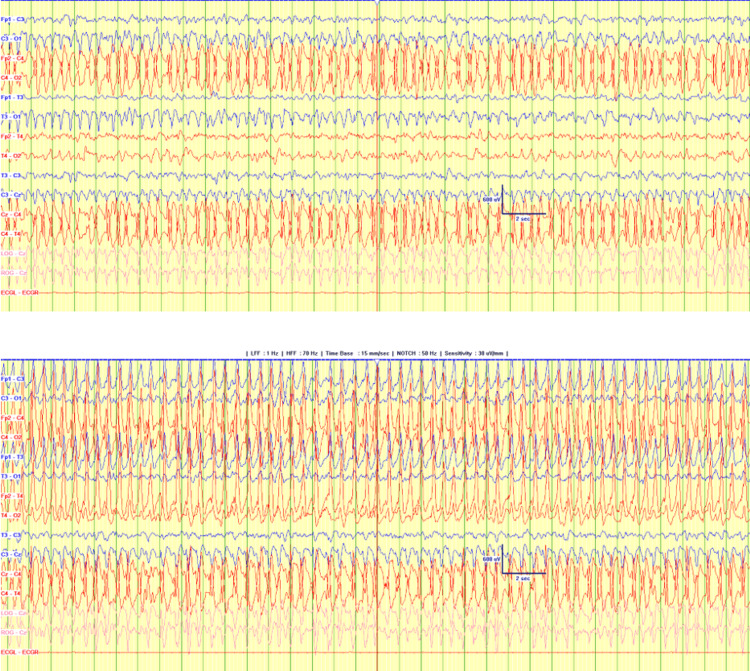
Continuous spike-and-wave activity involving the entire right hemisphere, exhibiting a waxing and waning pattern, likely represents markedly abnormal electrical activity superimposed with electrographic seizures.

Electroencephalography (EEG) demonstrated nearly continuous spike-and-wave activity involving the entire right hemisphere, exhibiting a waxing and waning pattern, likely represents markedly abnormal electrical activity superimposed with electrographic seizures (Figure 5).

Additional evaluations, including karyotyping, metabolic screening, and skeletal survey, were unremarkable. Echocardiography identified a moderate patent ductus arteriosus (PDA).

After initial seizure control with phenobarbitone, the seizures recurred. Levetiracetam was introduced (loading dose of 60 mg/kg followed by maintenance of 40 mg/kg/day), achieving partial control. A two-week follow-up EEG revealed improvement in background activity; however, intermittent clinical and subclinical seizures persisted, arising from the right posterior temporal and occipital regions. The phenobarbitone dose was then optimized to 8 mg/kg/day.

Based on the constellation of cutaneous findings, neuroimaging abnormalities, and Wood's lamp enhancement of skin lesions, a diagnosis of hypomelanosis of Ito (HI) was established. Subsequently, results from rapid whole-genome sequencing became available and were negative for pathogenic variants.

The neonate demonstrated steady clinical progress and was able to tolerate full oral feeds by the end of the third week of hospitalization. Despite this improvement, she continued to experience brief, intermittent clonic jerks of the left lower limb, without any associated changes in her vital signs or overall condition. Consequently, she was discharged on anti-seizure therapy consisting of levetiracetam (60 mg/kg/day) and phenobarbitone (8 mg/kg/day), with plans for multidisciplinary follow-up to address her comorbidities in the outpatient setting. At the two-month follow-up, oxcarbazepine was introduced (titrated up to 40 mg/kg/day) to control the persistent seizure episodes, resulting in noticeable clinical improvement.

## Discussion

Hypomelanosis of Ito is the third most common neurocutaneous syndrome, following neurofibromatosis and tuberous sclerosis complex [5]. The exact prevalence of hypomelanosis of Ito (HI) remains unknown; however, some studies estimate an incidence of approximately one in 3000 individuals [1], while others report rates as low as one in 8000-10,000 hospital visits [4,5].

While the exact pathogenesis remains incompletely understood, mosaic pathogenic variants in the mTOR gene have been identified in some cases presenting with brain overgrowth [2].

The hallmark clinical feature of HI is the presence of hypopigmented skin lesions distributed along the lines of Blaschko. These lesions often present as linear streaks, whorls, or patches and may be evident at birth or emerge later during childhood, particularly in individuals with lighter skin tones. They are most commonly located on the trunk, occasionally extending to the extremities, and rarely involve the face or scalp. Palms, soles, and mucous membranes are typically spared [6]. Moreover, no gender predilection has been reported [7].

HI demonstrates wide phenotypic variability, largely depending on the timing of genetic defects during embryonic development. Nonetheless, cutaneous hypopigmentation remains the only consistent clinical feature [7]. Extracutaneous manifestations commonly involve the central nervous system (CNS) and musculoskeletal system, with reported involvement in 33% to 94% of cases [1,8]. Additional anomalies may include ophthalmologic, cardiac, vertebral, and hair abnormalities. Neurological involvement is frequently reported and includes intellectual disability (most common), seizures, microcephaly, hypotonia, hydrocephalus, ataxia, speech delay, and sensorineural hearing loss [9]. Seizures often begin within the first few months of life and are attributed to underlying neuronal migration disorders such as polymicrogyria, hemimegalencephaly, and intracranial arteriovenous malformations [6].

Our patient's clinical presentation was consistent with the aforementioned neurological profile. She exhibited left focal seizures requiring polytherapy with anti-seizure medications, profound hypotonia, and significant feeding difficulties. MRI of the brain revealed bilateral sylvian polymicrogyria, heterotopia, lissencephaly, absence of the septum pellucidum, and a hypoplastic corpus callosum.

Diagnosis of HI is primarily clinical, based on characteristic skin findings, associated systemic involvement, and supportive neuroimaging [3]. In 1992, Ruiz-Maldonado et al. proposed clinical diagnostic criteria for HI, which continue to provide a useful framework for clinicians. A definitive diagnosis is established when this cutaneous presentation is accompanied by either one or more major criteria, namely, anomalies of the nervous or musculoskeletal systems, or by two or more minor criteria, which include congenital malformations unrelated to the nervous or musculoskeletal systems or chromosomal abnormalities. In cases where only the skin findings are present, or when they are associated with a single minor criterion, a presumptive diagnosis may be considered [3]. Histopathologic evaluation has limited diagnostic utility due to nonspecific findings and a lack of consistent histological variation [3].

The differential diagnosis for linear hypopigmented skin lesions includes systematized nevus depigmentosus, the fourth stage of incontinentia pigmenti, piebaldism, Waardenburg syndrome, and segmental vitiligo [4].

Currently, no curative treatment exists for HI. Management is largely supportive and multidisciplinary, with an emphasis on early recognition and targeted intervention for extracutaneous complications [4]. In our patient, seizure control remains a significant challenge, with partial response to a regimen of three anti-seizure medications. Cardiac evaluation revealed a moderate patent ductus arteriosus (PDA) with mildly reduced cardiac function. Other systemic evaluations, including metabolic, skeletal, and renal screening, were unremarkable. At two months of age, the patient has demonstrated improved feeding ability but continues to exhibit generalized hypotonia.

## Conclusions

Hypomelanosis of Ito is a rare but clinically important neurocutaneous disorder with marked phenotypic heterogeneity. Early recognition is essential given its frequent association with multisystem involvement, particularly central nervous system abnormalities. We present one of the earliest neonatal cases, characterized by unilateral Blaschko-linear hypopigmentation, ipsilateral hemihypertrophy, and early-onset focal seizures accompanied by extensive cortical malformations. This case reinforces that cutaneous mosaicism may serve as an early diagnostic clue, prompting timely neuroimaging and mitigating delays in identifying underlying neurodevelopmental abnormalities. Prompt multidisciplinary evaluation is essential to identify the extent of systemic involvement, guide individualized management, and optimize neurodevelopmental outcomes.
